# F62. RISKY DECISION-MAKING PERFORMANCE IN PATIENTS WITH EARLY SCHIZOPHRENIA-SPECTRUM DISORDER

**DOI:** 10.1093/schbul/sby017.593

**Published:** 2018-04-01

**Authors:** Sin Ki Luk, Tatia M C Lee, Eric YH Chen, Wing Chung Chang

**Affiliations:** The University of Hong Kong

## Abstract

**Background:**

Dysfunction in risky decision-making has been regarded as one potential contributing factor to functional impairment exhibited in patients with schizophrenia. Literature has revealed suboptimal risky decision-making in chronic schizophrenia patients. However, abnormality in risky decision-making has not been investigated in the early stage of illness. This study aimed to examine whether early schizophrenia patients displayed aberrant risky decision-making using two well-validated paradigms including Balloon Analogue Risk Task (BART; Lejuez et al., 2002) and Risky-Gains Task (RGT; Paulus et al., 2003).

**Methods:**

Thirty-three clinically-stable patients diagnosed with DSM-V schizophrenia-spectrum disorder (including schizophrenia, schizoaffective disorder or schizophreniform disorder) were recruited from specialized early intervention service for psychosis in Hong Kong. A group of healthy controls (n=32), matched with age, gender and educational levels, was enrolled for comparison. All participants were evaluated with a brief battery of cognitive assessment and two computerized risky decision-making tasks. Symptom assessment was also conducted for patients.

**Results:**

In both BART and RGT, patients with early schizophrenia-spectrum disorder performed worse than healthy controls regarding total points gained and reaction time. In BART, patients had significantly lower adjusted scores (F(1,63)=7.8, p<0.05) and lower balloon exploration rates than controls (F(1,63)=11.5, p<0.001), indicating that patients exhibited a tendency toward risk-aversion. In GRT, three-way analysis of variance revealed significant group x response interaction (F(1,63)=7.8, p<0.05), with post-hoc independent t-test showing that patients significantly preferred safe over risky options than controls (t=2.6, p<0.05). There were no significant correlations of risky decision-making parameters with symptom ratings and cognitive functions.

**Discussion:**

We extend previous findings of chronic samples to patients with early schizophrenia-spectrum disorder and indicate that suboptimal risky decision-making with risk-aversion preference has also been observed in the early course of illness. Further research is warranted to clarify the longitudinal change of aberrant risk-aversive behavioral patterns and its relationship with prospective functional and symptom outcomes.

